# Auxin production and plant growth promotion by *Microbacterium albopurpureum* sp. nov. from the rhizoplane of leafless *Chiloschista parishii* Seidenf. orchid

**DOI:** 10.3389/fpls.2024.1360828

**Published:** 2024-03-15

**Authors:** Elena A. Tsavkelova, Elena A. Volynchikova, Natalia V. Potekhina, Konstantin V. Lavrov, Alexander N. Avtukh

**Affiliations:** ^1^ Department of Biology, Shenzhen Moscow State University and Beijing Institute of Technology (MSU-BIT) University, Shenzhen, Guangdong, China; ^2^ Faculty of Biology, Lomonosov Moscow State University, Moscow, Russia; ^3^ Genomic Center of National Research Centre (NRC) “Kurchatov Institute”, Moscow, Russia; ^4^ Skryabin Institute of Biochemistry and Physiology of Microorganisms, Pushchino Scientific Center for Biological Research, Russian Academy of Sciences, Pushchino, Russia

**Keywords:** Actinobacteria, plant-associated *Microbacterium*, PGPB, microbial auxins, phytostimulation, indole-3-acetic and indole-3-pyruvic acids

## Abstract

The strains of the genus *Microbacterium*, with more than 150 species, inhabit diverse environments; plant-associated bacteria reveal their plant growth-promoting activities due to a number of beneficial characteristics. Through the performance of diverse techniques and methods, including isolation of a novel *Microbacterium* strain from the aerial roots of leafless epiphytic orchid, *Chiloschista parishii* Seidenf., its morphological and biochemical characterization, chemotaxonomy, phylogenetic and genome analysis, as well as bioassays and estimation of its auxin production capacity, a novel strain of ET2^T^ is described. Despite that it shared 16S rRNA gene sequence similarity of 99.79% with *Microbacterium kunmingense* JXJ CY 27-2^T^, so they formed a monophyletic group on phylogenetic trees, the two strains showed clear divergence of their genome sequences. The average nucleotide identity (ANI), average amino acid identity (AAI) and digital DNA-DNA hybridization (dDDH) values of ET2^T^ differed greatly from phylogenetically close JXJ CY 27-2^T^. Based on the differences being below the threshold for species similarity, together with the unique chemotaxonomic characteristics, strain ET2^T^ represents a novel species of the genus *Microbacterium*. Several genes, putatively involved in auxin biosynthesis were predicted. This strain revealed obvious plant growth-promoting activities, including diazotrophy and biosynthesis of tryptophan-dependent auxins (indole-3-acetic and indole-3-pyruvic acids). Microbial auxins directly stimulated the rhizogenesis, so that the ET2^T^-inoculated seeds of wheat, cucumber and garden cress showed evident promotion in their growth and development, both under optimal and under cold stress conditions. Based on phenotypic, chemotypic and genotypic evidences, the strain ET2^T^ belongs to the genus *Microbacterium*, order Micrococcales, class Actinomycetes, and it represents a novel species, for which the name *Microbacterium albopurpureum* sp. nov. is proposed, with strain ET2^T^ (VKPM Ac-2212, VKM Ас-2998) as the type strain.

## Introduction

The genus *Microbacterium* with *Microbacterium lacticum* as the type species belongs to phylum Actinomycetota, class Actinomycetes, order Micrococcales, and family Microbacteriaceae. It was originally proposed by Orla-Jensen (1919) to describe mesophilic or psychrophilic, aerobic, Gram-positive, non-spore-forming bacteria. The initial description was emended by Collins et al. (1983) and by Takeuchi and Hatano (1998). The genus is currently comprising 156 characterized species with 134 validly published (https://lpsn.dsmz.de/genus/microbacterium).

The cell morphology within the genus *Microbacterium* varies from coccoid to small irregular rods and branched fragmenting hyphae (Evtushenko and Takeuchi, 2006). These bacteria colonize diverse terrestrial and aquatic habitats, and they form associations with various plants and insects. They are isolated from fermented food, dairy products and clinical samples, and oil-containing soils and wastewater.

Actinomycetota is one of the dominant and well-characterized phyla among soil bacteria. The studies of 50 publicly available genome sequences of *Microbacterium* strains (https://www.ncbi.nlm.nih.gov/genome/browse#!/prokaryotes/Microbacterium) revealed that a type strain, *Microbacterium lacticum* DSM 20427, possesses a genome of 3.09 Mb and DNA G + C content of 70.3%, whereas *Microbacterium amyloliticum* DSM 24221 and *M. kyungheense* DSM 105492 have the 2.59-Mb- and 4.28-Mb-sized genomes and 64.56% and 70.70% Guanin-Cytosin (GC)-content, respectively. The representatives of family Microbacteriaceae contain the group B-type peptidoglycan with varying diamino acids (lysine or ornithine); their distinctive trait is a significant number of unsaturated respiratory menaquinones with 9 to 14 isoprene units and large amount of saturated iso- and anteiso-methyl branched fatty acids (Evtushenko and Takeuchi, 2006).

Bacteria of class Actinomycetes are commonly reported as plant-associated bacteria. Plant growth-promoting (PGP) strains of *Microbacterium* are reported as producers of the key plant growth stimulator, auxin (indoleacetic acid); they also exhibit 1-aminocyclopropane-1-carboxylate (ACC) deaminase activity, which plays the leading role in decreasing the level of the ethylene stress in plants (Madhaiyan et al., 2010). PGP activity of *Microbacterium* spp. also involves an ability to solubilize both insoluble inorganic (calcium phosphate) and organic phosphate (Zhang et al., 2017) as well as to produce volatile organic compounds (Cordovez et al., 2018). *Microbacterium endophyticum* and *M. halimionae* were described as endophytes of the salt-marsh plant *Halimione portulacoides*, providing an insight on their tolerance to salt stress (Alves et al., 2014). Antimicrobials produced by *Microbacterium* species reduce root infections of certain phytopathogenic fungi, such as *Rhizoctonia solani*, *Botrytis cinerea*, or *Fusarium verticillioides* (Barnett et al., 2006; Pereira et al., 2007); *Microbacterium nematophilum* and *M. maritypicum* effectively reduce the number of plant-pathogenic nematodes (Gravato-Nobre et al., 2005; Zhao et al., 2019). The abovementioned beneficial characteristics place *Microbacterium* strains among the most preferable plant-associated colonizers and PGP bacteria.

In our previous studies, we reported that wild-grown (Tsavkelova et al., 2007a) as well as greenhouse (Tsavkelova et al., 2003; Tsavkelova et al., 2016) tropical orchids formed tight associations with bacterial communities. Bacterial abundance and diversity of the dominant microbial groups were similar enough between different epiphytic plant hosts. However, microbial populations differed more significantly depending on the ecological environment of the orchid, varying between terrestrial versus epiphytic plants or between different microniches occupied by root-associated bacteria (aerial versus underground roots of the same epiphytic plant). For instance, the strains of *Rhizobium*, *Bacillus*, *Streptomyces*, *Enterobacter*, and *Pseudomonas* spp. were common for the substrate roots, which grow down into the soil or other substrate, whereas *Microbacterirum*, *Flavobacterium*, *Paenibacillus*, and *Sphingomonas* spp. were often isolated from the aerial roots of the epiphytes (Tsavkelova et al., 2007a, Tsavkelova et al., 2007b). Beneficial orchid-associated microorganisms, such as phototrophic cyanobacteria (Tsavkelova et al., 2003, Tsavkelova et al., 2022) and heterotrophic bacteria, colonizing the surface and the inner tissues of roots, often demonstrate direct plant-growth promoting activities, including nitrogen fixation and auxin [indole-3-acetic acid (IAA)] production (Tsavkelova et al., 2007a, Tsavkelova et al., 2007b, Tsavkelova et al., 2016). *Microbacterium* sp. D-23 strain, isolated from the aerial roots of *Dendrobium moschatum* (Buch.–Ham.) Swartz., showed tryptophan-depended IAA biosynthesis [with indole-3-pyruvic acid (IPA) and indole-3-acetaldehyde (IAAld) as intermediates specific for IPA pathway of auxin biosynthesis] (Tsavkelova et al., 2007b).

A search for bacteria prominent in plant growth and adaptation is a crucial aim of the modern biotech agronomy, considering the constantly progressing challenges of climate changing and anthropogenic impact across the Earth. Hence, the plant-associated microorganisms with an ability of surviving under different stress conditions, including extreme temperatures, flooding or drought, nutrient deficiency, or salt and heavy metal stresses, are of the primary interest. PGP bacteria (PGPB) are usually considered as root-associated colonizers, although the term PGP rhizobacteria (PGPR) is less usable nowadays, because beneficial bacteria may occupy other plant-related econiches, such as phyllosphere (stems and leaves), gemmisphere (buds and flowers), or carposphere (fruits and berries), settling on or within the plant tissues (Hallmann et al., 1997; Lodewyckx et al., 2002).

One of the effective approaches to discover novel microbes with intensified beneficial traits is to analyze the microbial populations of the plant hosts tolerant to harsh environmental conditions and resistant to lack of water or nutrients, hypermineralization, or high UV radiation. Such extreme environments may favor selection of beneficial PGPB helpful in alleviation of host-plant abiotic stresses (Marasco et al., 2012; Soussi et al., 2016; Niu et al., 2018; Gaete et al., 2020).

In this study, we report on a PGP strain of the genus *Microbacterium* isolated from the aerial roots of a rare leafless orchid *Chiloschista parishii* Seidenf. With the reduced stem and scale-looking leaves, this small epiphytic plant totally depends on its photosynthetically active aerial roots (Teoh, 2021) as well as on the functional and stable microbial community, which could provide a better adaptation to such a specific environment (Tsavkelova et al., 2022).

We describe strain ET2^T^ (= VKPM Ac-2212 = VKM Ас-2998) to be a novel species based on the genomic and chemotaxonomic analyses that showed its clear distinction from the rest of the *Microbacterium* species. The strain ET2^T^ produces plant growth stimulators that promote growth and development of different crops and herbs (wheat, cucumber, and garden cress). White to purple pigmentation of the microbial colonies stands as a distinctive feature of the species, which differs from the yellowish color of the other *Microbacterium* spp. The aim of this study was to clarify the taxonomic position of the novel strain ET2^T^ (=VKPM Ac-2212 = VKM Ас-2998) and to study its plant growth-promoting capacity. We propose to establish this strain as a novel species of the genus *Microbacterium* with the name *Microbacterium albopurpureum* sp. nov.

## Materials and methods

### Isolation and cultivation

Bacterial strain was isolated from the roots of a leafless epiphytic orchid of tribe Vandeae, *Chiloschista parishii* Seidenf. It is a monopodial perennial herb with reduced to minute-scale leaves and a short stem of only 5-mm to 12-mm long. Its aerial roots are radially directed. The 6-year-old plant is cultivated on a piece of bark under the natural light-dark cycle and the relative humidity of about 80% in the Greenhouse of the N.V. Tsitsin Main Botanical Garden of the Russian Academy of Sciences (Moscow, Russia). The temperatures are 27°C ± 2°C and 21°C ± 2°C in the daytime, and 21°C ± 2°C and 19°C ± 2°C in the nighttime for the summer and winter periods, respectively.

For the bacterial isolation, the collected aerial roots were intensively washed and rinsed with sterile tap water (STW) on a rotary shaker at 180 rpm for 5 min. The roots were then grounded with pestle in STW to prepare the initial suspension. The aliquot of 0.05 mL was plated onto the surface of R2A Agar (Difco) supplemented with nystatin (50 mg/mL) to prevent the fungal growth. The plates were incubated at 28°C for 5 to 7 days; single colonies were then isolated, and the pure culture was maintained on R2A medium.

For the co-cultivation in “neighbor culture” technique, imitating the competitive conditions with other bacteria, strain ET2^T^ was cultivated together with *Paenibacillus polymyxa* ET3 (VKPM B-14461) on the solid R2A medium at 30°C.

The strain ET2^T^ was stored on R2A agar plates and slants as well as lyophilized with skimmed milk and glucose solution. The strain is deposited in the Russian National Collection of Industrial Microorganisms (VKPM) under VKPM Ac-2212 number and in the All Russian Collection of Microorganisms (VKM) at the Skryabin Institute of Biochemistry and Physiology of Microorganisms, RAS (www.vkm.ru), under VKM Ас-2998 number.

### Phylogenetic characteristics and genome analysis

Genome of the strain ET2^T^ (=VKPM Ac-2212 = VKM Ас-2998) was recently sequenced *de novo* with a MinION device using Oxford Nanopore Technologies followed by polishing with Illumina reads, obtained using Illumina NovaSeq 6000 platform (Illumina, USA). Functional annotation was achieved using RAST online service (https://rast.nmpdr.org/) and submitted into GenBank (assembly accession number: CP128170; Volynchikova et al., 2024).

The 16S rRNA gene sequence was extracted from the complete genome sequence. It was compared with those of the *Microbacterium* type species using BLAST (https://blast.ncbi.nlm.nih.gov/Blast.cgi) and EzBioCloud databases (www.ezbiocloud.net). Relevant type sequences were aligned with ClustalW program (Thompson et al., 2003) and used for construction of phylogenetic trees in MEGA6 (Tamura et al., 2013).

A phylogenetic tree was constructed using the Neighbor-Joining (NJ) method, and evolutionary distances were calculated with the general time-reversible model (Zwickl and Holder, 2004) with *Arthrobacter* spp. as an outgroup. Two additional algorithms of Maximum Parsimony (MP) and Maximum Likelihood (ML) method based on the Tamura-Nei model were also used. A bootstrap analysis was performed with 1,000 replications to estimate the reliability of the tree topology.

Genomes of 12 closely related *Microbacterium* species were obtained from the NCBI database. Phylogenomic tree was inferred with the UBCG software based on the comparison of the 92 core genes (Na et al., 2018). Average nucleotide identity (ANI) values were calculated with JSpecies WS online program (https://jspecies.ribohost.com/jspeciesws/#analyse), average amino acid identity (AAI) values were calculated with AAI calculator (http://enve-omics.ce.gatech.edu/aai/), and *in silico* digital DNA-DNA hybridization (dDDH) values were calculated with Type (Strain) Genome Server (TSGS, https://tygs.dsmz.de/).

### Morphological, physiological, and biochemical characteristics

The strain ET2^T^ (= VKPM Ac-2212 = VKM Ас-2998) was cultivated for 3 to 5 days (young cultures) or up to a month or longer (old cultures) on R2A (Difco), tryptic soy broth (TSB, Merck), and TSB with agar (TSA, Merck) media. Unless stated otherwise, the cultures were incubated at 28°C for 3 days followed by cultivation at room temperature. Cell morphology was studied with scanning electron microscopy (JSM-6380LA, Jeol, Japan) at the User Facilities Center “Electron microscopy in life sciences” at Lomonosov Moscow State University. For this, the microbial cultures cultivated for 3 days in liquid TSB or solid R2A media, as well as those cultivated for 1.5 month on R2A, were fixed, dehydrated, dried by critical point in an HCP-2 (Hitachi Ltd., Japan), coated with Au–Pd (Eiko IB-3 Ion Coater, Hitachi, Japan), and analyzed under high vacuum at a pressure of 3 × 10^−5^ Torr with 20-kV accelerating voltage and SEI mode. Motility was determined under light microscope at ×1,500 magnification by the hanging drop technique.

Catalase activity was examined by the appearance of bubbles of O_2_ in a 3% (v/v) H_2_O_2_ solution. Acid production from carbohydrates was examined in a medium containing peptone (4 g/L), K_2_HPO_4_ (1.0 g/L), NaCl (2.0 g/L), 0.2% solution of bromocresol purple, and the tested carbon source (5 g/L; pH 7.0–7.2).

Growth at different temperatures (20°C, 30°C, 37°C, and 55°C), pH (5.0, 6.0, 6.5, 7.0, 8.0, 9.0, and 10.0), and salinity (2%, 5%, and 10% w/v) was assessed in liquid TSB (Merck) medium by monitoring the increase in optical density at 540 nm (OD_540_) value of the culture. The ability of nitrogen-fixing (diazotrophy) activity was determined by cultivation on semisolid and liquid nitrogen-free media of Fedorov–Kalininskaya with sucrose as a carbohydrate. Nitrate reduction and hydrolysis of starch, gelatin, and casein were determined as described by Cowan (1974).

Antibiotic susceptibility was determined according to the disc diffusion susceptibility test procedure on R2A plates at 25°C using antibiotic discs (NICF, Russia) containing (μg per disc): ampicillin (10), tetracycline (50), and cefalexin (30). Bacterial susceptibility was estimated on the basis of diameter of the growth inhibition zone around each disc.

### Chemotaxonomy

To determine the chemotaxonomic traits of the strain ET2^T^ (= VKPM Ac-2212 = VKM Ас-2998), it was cultivated in broth medium, containing peptone (5 g/L), K_2_HPO_4_ (0.2 g/L), yeast extract (3.0 g/L), and glucose (5 g/L) for 72 h on a rotary shaker (180 rpm) at 28°C. The cells were then centrifuged and washed three times in 0.9% NaCl solution followed by liophilization. Purification of the cell walls, extraction of the glycopolymers, and determination of the cell-wall sugars were performed as described elsewhere (Potekhina et al., 2011). To obtain the peptidoglycans, carbohydrate-containing polymers were removed from the cell walls (30 mg) by extraction with 5% trichloroacetic acid at 100°C for 20 min. A 10 mL of trypsin solution (Tris-HCl buffer at 1 mg/mL, pH 7.85) was added to the remains of the cell walls, and enzyme treatment was carried out at 37°C for 20 h. The obtained sample was repeatedly washed with Tris-HCl buffer and then treated with 4% sodium dodecyl sulfate (SDS) in the same buffer at 100°C for 5 min. After SDS was washed, the peptidoglycans were lyophilized.

To determine the amino acid composition of the peptidoglycans, the samples were hydrolyzed (freshly prepared concentrated hydrochloric and trifluoroacetic acids in a 2:1 ratio with the addition of 0.1% β-mercaptoethanol, 1 h at 155°С) and then analyzed with L-8800 amino acid analyzer (Hitachi, Tokyo, Japan) according to the method described by Trofimova et al. (2016).

To determine the composition of cell-wall sugars, acid hydrolysis was performed with 2 M HCl for 3 h at 100°C; the products of hydrolysis were separated by paper chromatography as described previously (Potekhina et al., 2011).

The fatty acid composition was analyzed using a gas chromatograph-mass spectrometer 7890B+5977B (Agilent Technologies, USA). For that, the cell biomass was dried, saponificated (3.75 M NaOH/MeOH, 100°C, 30 min) and subjected to acidic methanolysis (6 N HCl/MeOH, 80°C, 10 min). The products of methanolysis were extracted with hexane:methyl tert-butyl ether (1:1 w/w) and processed with alkali (0.3 M NaOH, 5 min). The obtained products were separated on a 5% phenyl-methyl silicone capillary column HP-5MS (0.25 mm × 30 m) in the temperature gradient from 50°C to 300°C at 40°C min^−1^. Fatty acids and other lipid components were ionized by electron impact and analyzed in the scan mode. The compounds were identified using the NIST17 mass spectrometer library. Fatty acid content was determined as the percentage of the total ion current peak area (Sorokin et al., 2021).

Polar lipids were extracted from the freeze-dried cells with chloroform/methanol mixture (2:1, by vol.) and separated on the HPTLC Silica gel 60F plates (Merck, Germany) using the following solvent systems: chloroform/methanol/water (65:25:4, by vol.) in the horizontal dimension and chloroform/methanol/acetic acid/water (80:12:15:5, by vol.) in the vertical dimension for two-dimensional TLC (Minnikin et al., 1984). Polar lipids were identified using sprayed/aerosol reagents for visualizing the spots: molybdenum blue for phospholipids, ninhydrin for aminolipids, and alpha-naphthol solution for glycolipids (Minnikin et al., 1984; Dittmer and Lester, 1964).

### Microbial auxin production and TLC analysis

For determination of auxin production, the following liquid nitrogen-limiting medium was used: glucose, 5.0 g/L; K_2_HPO_4_, 0.5 g/L; KH_2_PO_4_, 0.3 g/L; MgSO_4_·7H_2_O, 0.1 g/L; NaCl, 0.5 g/L; CaCl_2_·6H_2_O, 0.03 g/L; yeast extract, 0.5 g/L; pH 7.0–7.2. The medium was supplemented with L-tryptophan (400 mg/L). The cultivation was performed in 50 mL of media on the rotary shaker (180 rpm) at 30°C in darkness. Aliquots of the culture media were sampled for indole extraction and analysis 72 h after inoculation.

Relative content of auxins was estimated with the Salkowski reagent (Gordon and Weber, 1951; Glickmann and Dessaux, 1995) as previously described (Tsavkelova et al., 2007a, Tsavkelova et al., 2016). After the reagent was added to the supernatants, the samples were incubated for 20 min at 25°C in the darkness. The development of pink color confirms the presence of IAA. For the relative quantitative estimation of the auxin content by colorimetric assay, optical density was measured at 530 nm, by using a standard curve produced from serial dilutions prepared from the 100 mM IAA stock solution.

For IAA presence and detection of its potential precursors, thin-layered chromatography was used. One milliliter of 3-day-old cultures was centrifuged; supernatant was collected, and pH was adjusted to 2.8–3.0. Aliquots of 800 µL were extracted with ethyl acetate (1 mL) by vigorous shaking for 10 min. After phase separation, the upper fraction was removed and evaporated, and the residue was dissolved in 30 µL of methanol. Silica gel “Macherey-Nagel GmbH&Co. KG” (Germany) plates with UV (254 nm) indicator were used. The samples and 1 mM standard solutions of IAA, indole-3-acetamide (IAM), and indole-3-pyruvic acid (IPyA) diluted in ethanol as well as 2 mM L-tryptophan were spotted onto the plates, followed by their developing in chlorophorm:ethyl acetate:formic acid (50:40:10) running solvent. Then, the plates were dried and visualized under UV light, followed by spraying with van Ehmann’s reagent (Ehmann, 1977) and heating to 90°C until the spots were colored. The R*f* and color of the visualized spots were compared with the standard compounds.

### Evaluation of microbial auxin activity by plant assay

The biological activity of auxins produced by *Microbacterium* ET2^T^ was estimated in a bioassay with the common bean (*Phaseolus vulgaris* L.) plant cuttings as a model for IAA-induced plant rhizogenesis, developed by R. Turetskaya in 1961 (Kefeli, 1978). The bean cuttings are susceptible to exogenous IAA, and the formation of the adventitious roots and their localization on the stem correlates with the amount of IAA, making visual the auxin effects in formation and growth of the new nascent roots along the stem of the cuttings. This bioassay is used as evidence for IAA-induced plant growth responses. For this aim, the germinating beans were soaked in tap water for 12 h. After the evident primary root emergence, the beans were transferred onto a colander-like rack in the water tank, allowing the roots to remain under the water. After 9–10 days of cultivation, the plants with well-developed unifoliate leaves were selected and the basal part (1 cm above the root crown) was cut off under water. The cuttings were submerged in bacterial culture supernatants for 6 h. The solutions of IAA (30 mg/L) and IPA as well as tap water were used as positive and negative controls, respectively (Tsavkelova et al., 2007a, Tsavkelova et al., 2016). The height of root emergence and root number were analyzed 10 days after the treatment.

### Plant growth-promoting capacity

To estimate the PGP potential of *Microbacterium* ET2^T^, seeds of wheat, cucumber, and garden cress, as well as different treatment approaches were used. Wheat (*Triticum aestivum* L.) seeds were preliminary soaked in water for 1 h and then soaked in either of the following solutions for 2 h: *i*) *Microbacterium* ET2^T^ suspension (OD_530 = _1.8; 1.6 × 10^9^ colony-forming units (CFU)/mL), cultivated for 4 days in N-free medium supplemented with L-tryptophan (400 mg/L) or *ii*) supernatant of the same culture resulted after its centrifugation for 5 min at 12,000 rpm; *iii*) tap water as a control.

Cucumber (*Cucumis sativus* L.) seeds were soaked for 2 h in the same treatments described above without preliminary soaking in water. A part of the seeds was consequently subjected to cold treatment (4°C) for 24 h to estimate bacterial influence under the stress conditions. Seeds of garden cress (*Lepidium sativum* L.) were flooded with bacterial culture after they were placed in vermiculite (Uralvermiculit, Russia). Six wheat seeds, four cucumber seeds, and 35–40 seeds of garden cress were placed per pot filled with vermiculite. The pots were cultivated at 28°C with day/night photoperiod of 12 h and watered daily. Plant growth parameters, including root weight, shoot length, and total weight of vegetative parts (stems with leaves) were measured 14–18 days after the treatment.

### Mining for putative genes involved in IAA production in ET2

For the search of the protein-coding genes, which could be potentially involved in IAA biosynthesis, two approaches were conducted *i)* by using protein functions in annotation of the whole-genome of ET2, and *ii*) by blasting sequences of bacterial IAA-related proteins as queries. The sequences of IAA-related genes were acquired using tBLASTn algorithm-based search in the translated nucleotide sequence of ET2 genome (parameters: e-value: 1e-5, coverage > 50%, identity > 30%). Query proteins were picked from UNIPROT and NCBI databases (accession numbers are provided in **Table 1**) by querying with protein names.

**Table 1:** Genome features and genome comparison, including average amino acid identity (AAI), average nucleotide identity (ANI), and *in silico* DNA-DNA hybridization (dDDH) between strain ET2^T^ and most closely related *Microbacterium* spp.

Characteristics	ET2	1^a^	2	3	4	5	6	7	8	9	10	11	12
GenBank accession no.	CP128170	JAJPDW010000000	CP044231	JAOPFX010000000	VRSV01000000	JAKNUU000000000	JAKNUS010000000	VFPS01000000	VRSW01000000	JAAGRY010000000	BKAH01000000	RCDB01000000	CP038266
Genome size (bp)	3,604,840	3,024,072	3,262,719	3,330,427	3,545,086	3,573,522	3,497,265	3,086,904	2,821,547	3,215,183	2,987,352	3,118,122	3,828,279
Number of contigs	1	18	1	5	2	7	28	9	12	147	31	7	1
N50 length (bp)	3,604,840	619,178	3,262,719	1,845,146	2,139,879	1,119,066	300,586	613,777	1,483,109	40,778	202,542	947,782	3,828,279
L50 lenth (bp)	1	2	1	1	1	2	5	2	1	26	5	2	1
Total gene number	3,490	2,927	3,072	3,101	3,351	3,321	3,493	3,073	2,601	3,175	2,874	2,988	3,750
Protein coding gene number	3,325	2,560	2,972	3,034	3,280	3,020	3,105	2,861	2,517	2,784	2,789	2,901	3,679
tRNA	48	46	48	46	46	48	50	47	50	46	47	48	45
rRNA	3	3	6	6	4	3	3	3	5	3	3	5	6
G+C content (mol%)	69.4	70.2	70.2	70.4	69.8	69.3	69.8	70.3	63.5	69.5	71.0	70.5	70.5
Completeness (%)	99.49	81.42	98.04	98.11	98.57	83.54	77.38	98.57	96.01	81.73	99.2	98.45	97.38
Contamination (%)	0.63	1.61	0.71	1.01	1.49	0.60	0.71	3.04	2.19	1.52	0.36	0.17	0.71
AAI (%)^b^	–	69.77	68.61	69.35	68.08	66.48	88.63	67.86	63.17	66.44	68.37	67.32	69.60
ANI (%)^c^	–	74.76	73.95	75.06	74.46	73.99	86.21	74.70	71.10	74.36	74.45	74.21	74.96
dDDH (%)^d^	–	19.7	19.4	19.9	19.8	19.5	32.6	20.2	20.1	19.8	19.5	19.9	20

a 1, *Microbacterium aurantiacum* GX14001 (Gao et al., 2022); 2, *Microbacterium caowuchunii* ST-M6^T^ (Tian et al., 2021); 3, *Microbacterium fluvii* IDR2; 4, *Microbacterium hatanonis* JCM14558^T^ (Bakir et al., 2008); 5, *Microbacterium invictum* DSM 19600^T^ (Vaz-Moreira et al., 2009); 6, *Microbacterium kunmingense* JXJ CY 27-2 (Xiao et al., 2022)^T^; 7, *Microbacterium lacticum* DSM20427^T^ (Bellassi et al., 2020); 8, *Microbacterium mitrae* M4-8^T^ (Kim et al., 2011); 9, *Microbacterium paulum* 2C^T^ (Bellassi et al., 2021); 10, *Microbacterium saccharophilum* K-1^T^ (Ohta et al., 2013); 11, *Microbacterium telephonicum* S2T63^T^ (Rahi et al., 2018); 12, *Microbacterium wangchenii* dk512^T^ (Dong et al., 2020). T, type strain of the species. All data were obtained from the NCBI Genome database.

b AAI was calculated using AAI calculator at http://enve-omics.ce.gatech.edu/aai/.

c ANIb based on BLAST+ was calculated at https://jspecies.ribohost.com/jspeciesws/#analyse.

d In silico DNA-DNA hybridization rates were calculated at https://tygs.dsmz.de/.

### Statistical analysis

All experiments were repeated no less than three to five times. For determination of microbial auxin amounts, five replicates were used. The kidney been bioassay was performed with five to six cuttings for each sample in triplicates. For the plant growth stimulation, four repetitions were used for each treatment. The mean values of repetitions were calculated ± standard deviation (SD). To evaluate the statistical difference, the values were separated by Student’s *t*-test (*P* ≤ 0.05).

## Results and discussion

### Phenotypic and cultural characteristics

**Figure 1:**
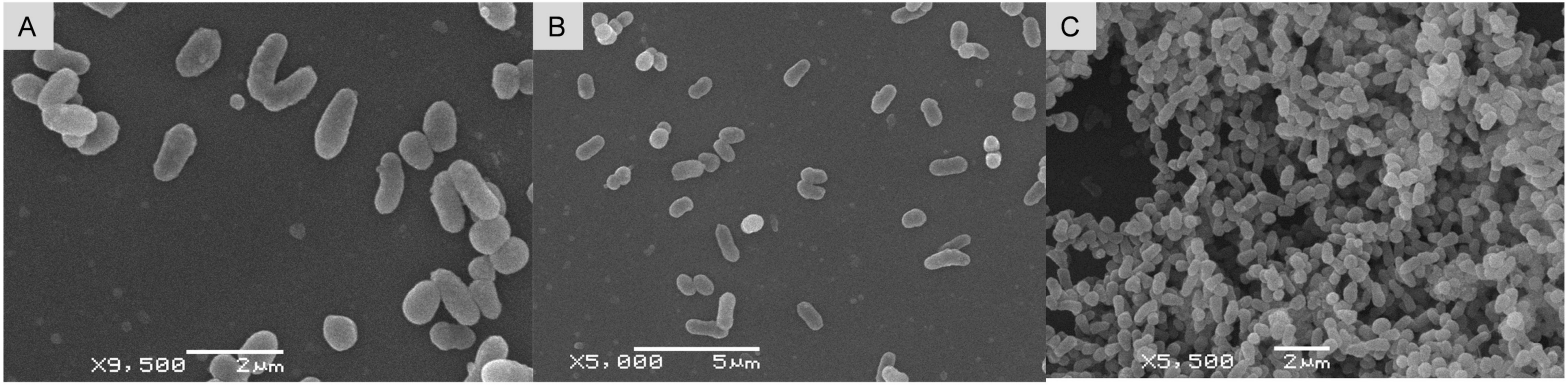
Scanning electron micrographs of *Microbacterium* strain ET2^T^ in 3-day-old **(A, B)** and 1-month-old **(C)** cultures, cultivated on R2A **(A, C)** and in liquid TSB **(B)**.

Strain ET2^T^ (= VKPM Ac-2212 = VKM Ас-2998) is aerobic, catalase-positive, non-motile, and non-spore-forming bacterium, forming short rods of 0.8-µm to 1.4-µm long and 0.3-µm to 0.5-µm wide (**Figure 1**). Cell morphology does not differ between young (3 days of cultivation) and old cultures. In addition, no morphological differences were observed between the bacterial cultures grown in different media (solid R2A and liquid TSB).

**Figure 2:**
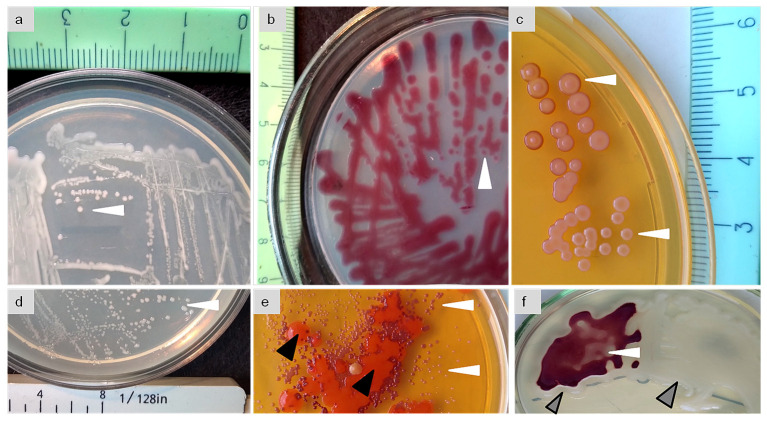
Phenotipic characteristic and pigmentation of the colonies (white arrows) of *Microbacterium* strain ET2^T^ (= VKPM Ac-2212 = VKM Ас-2998) grown in a pure culture **(A–D)** or together with alien bacterial cultures **(E, F)** as contaminated (**E**, black arrows) or co-cultured with *Paenibacillus polymyxa* (**F**, gray arrows).

Colonies are circular, more convex, and mucous on R2A and flatter with evident vertex on TSA. The main distinctive feature of the strain ET2^T^ is its pigmentation; the colonies are more whitish or slightly pink (**Figures 2A, D**), but they acquire bright purple color (**Figures 2B, C**) when becoming old (2–3 months after inoculation) or when other cultures are growing in close proximity (**Figures 2E, F**). Single colonies (**Figure 2E**) or the whole population acquire violet color nearby the alien bacterial colonies as shown with *Paenibacillus polymyxa* ET3 (**Figure 2F**).

Srain ET2^T^ demonstrates distinct alteration in its pigmentation, hinting its high competitiveness in relation to other microbes due to its secondary metabolite production. Together with the specificity of the aerial rhizoplane econiche, where we isolated it from, we suggested that the strain might be prominent in plant growth promotion.

### Phylogenetic analyses

**Figure 3:**
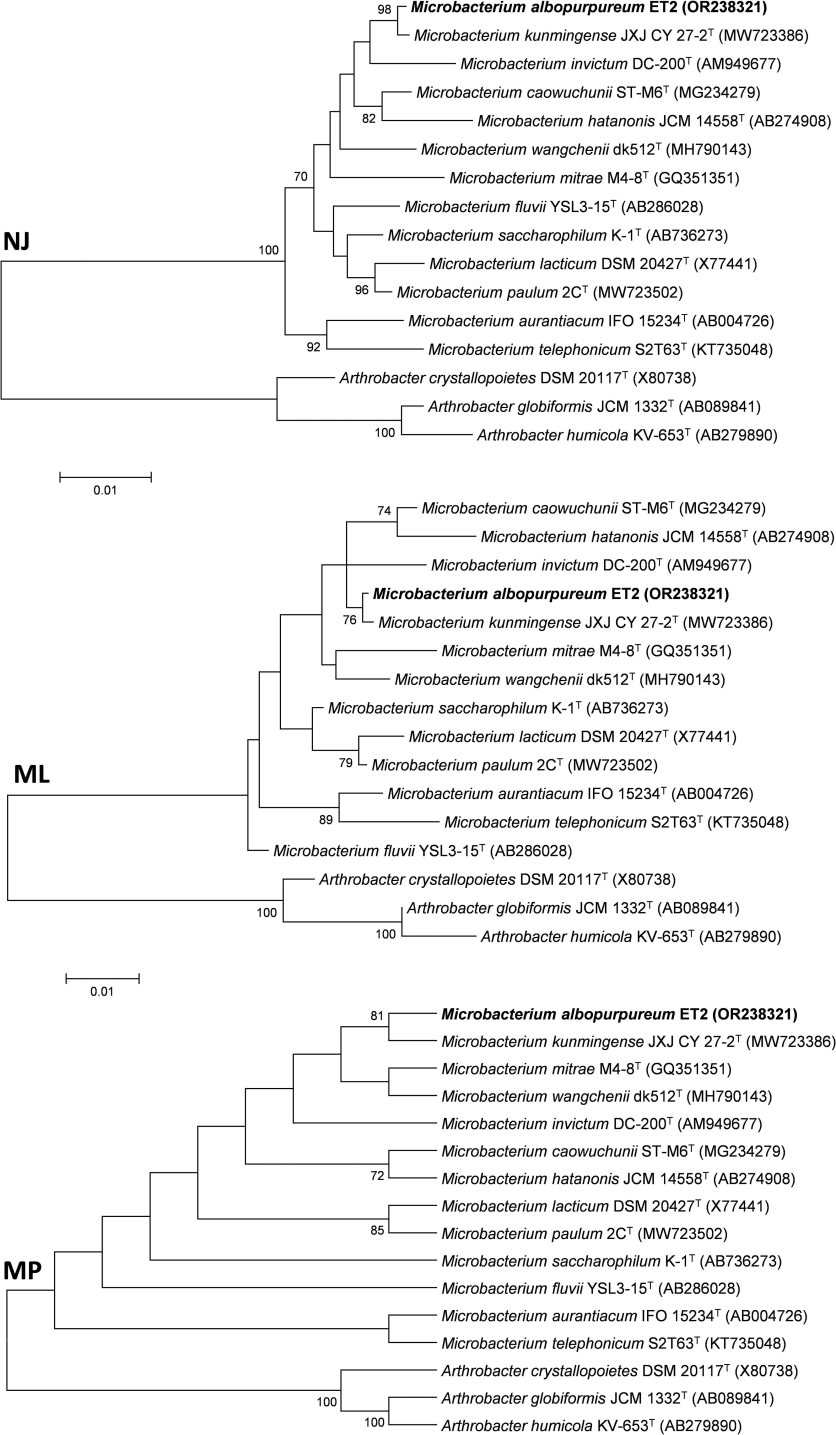
Phylogentic tree based on 16S rRNA gene analysis from ET2^T^ and the closest type strains of the genus *Microbacterium*. The tree was created with MEGA6 software using Neighbor Joining (NJ), Maximum Likelyhood (ML), and Maximum Parsimony algorithms. Numbers at the branching points show the bootstrap values (>70%) for 1,000 replications. GenBank accession numbers are provided in parentheses. Bar corresponds to 1 nucleotide substitution per 100 nt. *Arthrobacter* spp. were used as an outgroup. “T,” the type strain.

Comparative analysis of the 16S rRNA sequence of the strain ET2^T^ showed the highest similarity with *Microbacterium caowuchunii* ST-M6^T^ and *Microbacterium saccharophilum* K-1^T^, exhibiting similarity rates of 98.61% and 98.58%, respectively. Additional analysis performed in EZBioCloud database revealed a recently described (Xiao et al., 2022) and newly proposed (https://lpsn.dsmz.de) *Microbacterium kunmingense* JXJ CY 27-2^T^ as a strain with the highest similarity of 99.79%. Generally, a threshold set at 97% is used to distinguish between the species (Schloss and Handelsman, 2005). However, it is known that different bacterial species may share up to 99% similarity of their respective 16S rRNA sequences (Fox et al., 1992; Nguyen et al., 2016). To further investigate the position of ET2^T^ within the *Microbacterium* group, phylogenetic analysis was conducted by using different algorithms, including NJ, ML, and MP, which showed similar clusterization patterns of the strain ET2^T^ within *Microbacterium* genus. All three analyses (**Figure 3**) proposed a monophyletic groups consisting of ET2^T^ and *M. kunmigense* JXJ CY 27-2^T^ with the bootstrap values varying between 76 (ML), 81 (MP), and 98 (NJ). The other closely related species were *Microbacterium invictum* DC-200^T^, *M. caowuchunii* ST-M6^T^, *M. wangchenii* dk512^T^, *M. mitrae* M4-8^T^, and *M. hatanonis* JCM 14558^T^.

**Figure 4:**
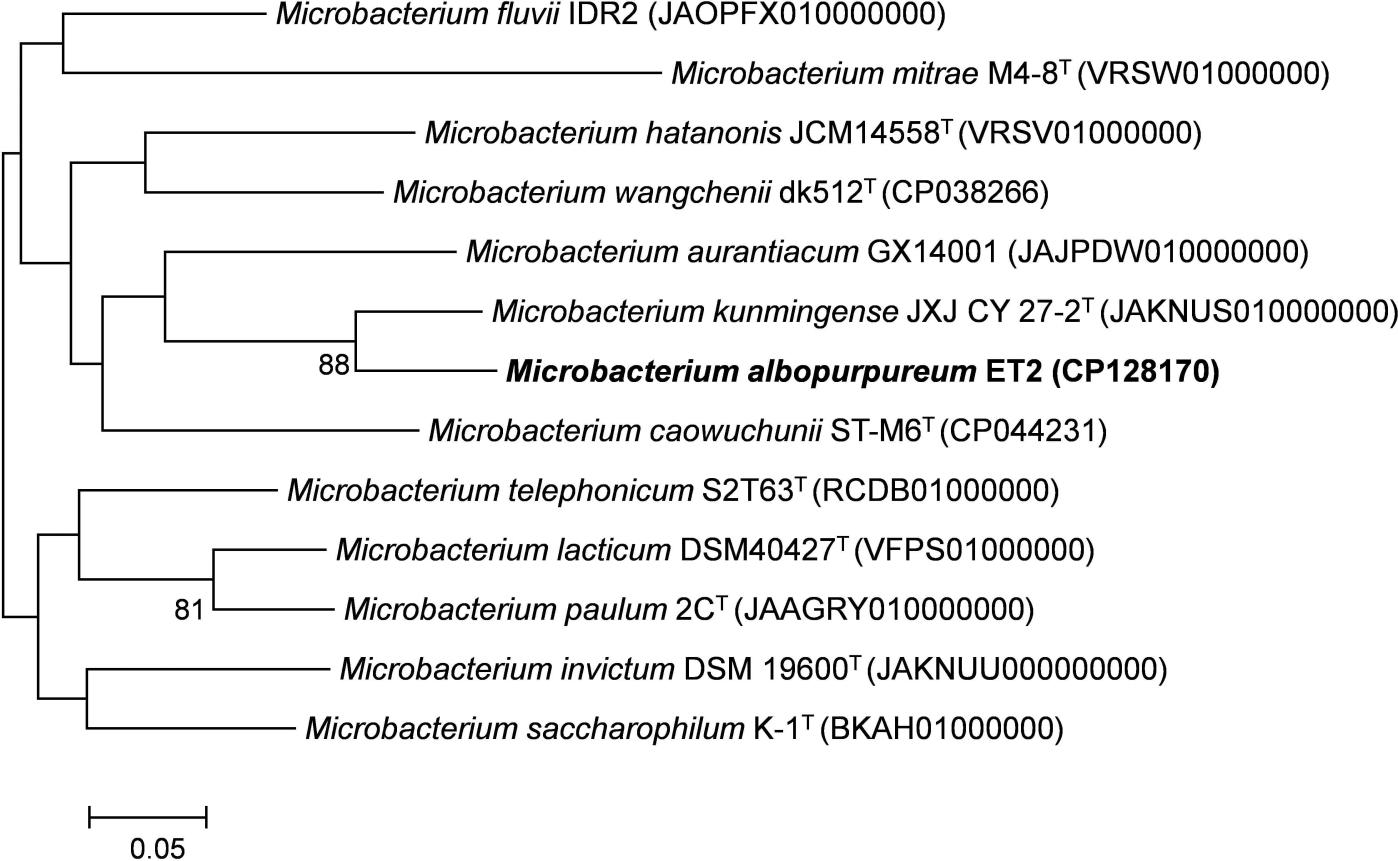
Phylogenomic tree based on 92 core genes showing relations of ET2^T^ with the closest *Microbacterium* spp. Numbers at the branching points show the gene support index (>70%). RefSeq accession numbers are provided in parentheses. Bar corresponds to 0.05 substitutions per nucleotide position. “T,” the type strain. The tree was rooted at the midpoint.

The genomic tree (**Figure 4**) also supported a separate monophyletic group formed by ET2^T^ and *M. kunmingense* JXJ CY 27-2^T^ as proposed by the 16S rRNA analysis. However, further genomic analyses of the strain showed that the *in silico* genome comparison values varied between 63.17% and 88.63% for the AAI, between 71.10% and 86.21% for the ANI, and between 19.4% and 32.6% for *in silico* dDDH analysis (**Table 2**). The obtained values are far below the thresholds used to distinguish between the species for any of the values being < 95% (AAI and ANI) and < 70% (dDDH) (Wayne et al., 1987; Goris et al., 2007; Kim et al., 2014; Menon et al., 2020), rejecting the hypothesis that the strain ET2 is a *M. kunmingense* or other *Microbacterium* species.

**Table 2:** Differential characteristics of strain ET2^T^ and phylogenetically related species of the genus *Microbacterium*.

Characteristic	ET2^T^	1	2	3	4	5	6	7
DNA G+C content (mol%)	69.4	69.8	70.1	70.4	66.5	69.0	71	73
Colony color	From white to purple	Yellow	White/yellow	Ivory	Yellow	Light yellow	White	Yellow
Growth temperature (^°^C)	25–55 W (30–37)	10–44 (32)	10–37	28	10–42	10–37	4–50 (28)	9–37
Growth pH	6.0–9.0 (7.0)	5.0–10.0 (7.0–8.0)	6.0–9.5	7.5	nd	6.0–9.0 (7.0)	5.0–10.0	6.0–11.0 (7.0)
5% NaCl tolerance (w/v)	+	+	+	−	−	−	+	−
Assimilation of: Glucose	**+**	+	+	+	W	+	+	+
Sucrose	**+**	+	−	+	nd	nd	nd	nd
Galactose	**+**	+	−	+	nd	nd	W	nd
D-mannose	**+**	+	+	+	+	+	+	+
D-maltose	**+**	+	+	+	+	+	nd	+
D-Mannitol	+	+	+	nd	+	+	+	+
Trisodium citrate	−	nd	nd	nd	−	−	+	−
Starch	W	+/−^a^	−	nd	nd	nd	nd	−
Gelatin	−	nd	+	nd	nd	−	−	+
Acid from glucose	W	nd	W	+	W	−	+	nd
Sucrose	+	nd	−	+	+	−	−	nd
Galactose	+	nd	W	+	nd	−	W	nd
D-mannose	+	nd	+	+	+	−	−	nd
D-maltose	+	−	−	+	+	+	+	nd
Catalase	+	+	+	+	+	+	+	+
Nitrate reduction	**−**	−	+	nd	**−**	**−**	**−**	**−**
Cell-wall diamino acid	Orn	Lys	Lys	Orn	Orn	Orn	Orn	Lys
Cell-wall sugars	Rha, Fuc, Glc, Gal	Man, Rib, Gal, Rha, Ara	nd	Rha, Rib, Man, Gal	6dTal, Man, Gal	Rha, Gal	Rib, Glc, Gal	Rha, Gal
Fatty acids (%)	Anteiso-C_15:0_ (35.35), iso-C_16_:_0_ (31.96), anteiso-C_17:0_ (22.78); iso-C_15:0_ (4.0), C_16:0_ (3.08), iso-C_17:0_ (1.80), iso-C_14:0_ (1.02)	Anteiso-C_17:0_ (43.4), anteiso-C_15:0_ (27.1), iso-C_17:0_ (9.6), iso-C_16:0_ (9.5), iso-C_15:0_ (6.8), C_16_:_0_ (2.5), iso-C_14:0_ (0.2)	Anteiso-C_15 : 0_ (52.0), anteiso-C_17 : 0_ (25.1), iso-C_16 : 0_ (9.7), iso-C_15:0_ (5.5), C_16:0_(3.5),iso-C_17:0_ (3.0), C_17:0_ (0.3), C_18:0_ (0.3), iso-C_14:0_ (0.3), iso-C_18:0_(0.2), anteiso-C_15:0_ (0.1)	Anteiso-C_15:0_ (48.6), anteiso-C_17:0_ (24.4), iso-C_16:0_ (12.1), iso-C_15:0_ (7.0)	Anteiso-C_15:0_ (55.0), anteiso-C_17:0_ (31.1), iso-C_16:0_ (11.3)	Anteiso-C_15:0_ (48.1), anteiso- C_17:0_ (34.7-36.6^a^), iso-C_16:0_ (10.5- 12.5^a^), anteiso-C_17:1_ (2.1), C_14:0_ (1.1)	Anteiso-C_15:0_ (38.5), C16:0 (18.9), anteiso-C_17:0_(17.1), iso-C_16:0_ (14.1), C_18:0_(6.0), C_14:0_ (2.8), iso-C_14:0_ (1.7), iso-C_15:0_ (1.0)	Anteiso-15:0 (61.65), anteiso-C_17:0_ (26.22), iso-C_16:0_ (7.30), iso-C_16 : 0_ (3.09)

Strain/species: strain ET2^T^ (VKPM Ac-2212 = VKM Ас-2998); 1, *M. kunmingense* JXJ CY 27-2^T^; 2, *M. invictum* DC-200^T^; 3, *М. сaowuchunii* ST-M6^T^; 4, *M. schleiferi* JCM 9175^T^; 5, *M. hatanonis* JCM 14558^T^; 6, *M. sediminis* ylb-01^T^; 7, *M. saccharophilum* K-1^T^.

Data for ET2^T^ were obtained in this study. Other data for the reference strains were taken from Kageyama et al. (2006); Bakir et al. (2008); Vaz-Moreira et al. (2009); Yu et al. (2013); Tian et al. (2021); Xiao et al. (2022), and Ohta et al. (2013);

a Data were taken from Kageyama et al. (2006) and Yu et al. (2013).

+, positive; −, negative; W, weakly positive; nd, no data. Orn, ornithine; Lys, lysine; Ara, arabinose; Fuc, fucose; Gal, galactose; Glc, glucose; Man, mannose; Rha, rhamnose; Rib, ribose; 6dTal, 6-deoxy-l-talose.

Data presented in **Table 2** clearly demonstrated the differences in genomic features between ET2^T^ and the strains of *Microbacterium aurantiacum* GX14001, *M. caowuchunii* ST-M6^T^, *M. fluvii* IDR2, *M. hatanonis* JCM14558^T^, *M. invictum* DSM 19600^T^, *M. kunmingense* JXJ CY 27-2^T^, *M. lacticum* DSM40427^T^, *M. mitrae* M4-8^T^, *M. paulum* 2C^T^, *M. saccharophilum* K-1^T^, *M. telephonicum* S2T63^T^, and *M. wangchenii* dk512^T^.

The complete genome of strain ET2 (CP128170) contains 1 contig with a total length of 3,604,840 bp versus 3,497,265 bp in *M. kunmingense* JXJ CY 27-2^T^ and a N50 length of 3,604,840 bp versus 300,586 bp, respectively. It has 3,325 versus 3,105 in *M. kunmingense* JXJ CY 27-2^T^ protein-coding genes; the difference in the DNA G + C content comprised 69.4% and 69.8%, respectively. In addition, phenotypic characteristics of *M. kunmingense* JXJ CY 27-2^T^ described by Xiao et al. (2022) diverge from those observed for strain ET2 as it is described below. Therefore, despite the seeming similarities between the strain ET2 and *M. kunmingense* JXJ CY 27-2^T^ postulated by the 16S rRNA gene-based analysis, whole genome-based analysis revealed the evident differences between the two strains.

### Physiological and biochemical characteristics

Growth of the strain ET2^T^ was observed in TSB at 25°C–55°C with optimum at 30°C–37°C and pH 6.0–9.0 (optimum, pH 7.0). Under NaCl supplementation, the growth was detected at 0%–5% up to 10% (w/v) of sodium chloride. The cells were positive for catalase activity and assimilation of glucose, sucrose, galactose, D-mannose, D-maltose, and D-mannitol. Weak positive reaction was shown for glycerol assimilation and starch hydrolysis. On the other hand, the strain was negative for nitrate reduction, utilization of trisodium citrate and pyruvate, as well as for the hydrolysis of gelatin and casein. Acid was produced from sucrose, galactose, D-mannose, D-maltose, and D-mannitol.

The cell-wall hydrolysates of ET2^T^ contained glucose and galactose as the main sugars and fucose with rhamnose as minor whole-cell sugars. Glycerol was also identified. The strain contained L-ornithine as the diagnostic amino acid along with alanine, valine, glutamic acid, and glycine in the peptidoglycan (in a ratio 1.0:1.8:0.8:0.4:3.5). The major fatty acids of strain ET2^T^ were anteiso-C_15:0_ (35.35%), iso-C_16:0_ (31.96%), and anteiso-C_17:0_ (22.78%); iso-C_15:0_ (4.0%), C_16:0_ (3.08%), iso-C_17:0_ (1.80%), and iso-C_14:0_ (1.02%) were detected as minor compounds. The polar lipid profile is typical for members within the genus *Microbacterium*. The strain ET2 contained phosphatidylglycerol and diphosphatidylglycerol as major components with trace amounts of a few unidentified glycolipids and an unidentified polar lipid. The strain was resistant to ampicillin (10 μg per disc) and cefalexin (30 µg) and showed intermediate susceptibility to tetracycline (50 μg) with inhibition zones varying between 15 mm and 17 mm.

The results of comparative analysis based on the deferential traits of the strain ET2^T^ and the phylogenetically closest species of the genus *Microbacterium* were summarized in **Table 3**. In addition to its specific color, strain ET2^T^ differs from other *Microbacterium* strains by a more thermophilic lifestyle. Salinity tolerance of ET2^T^ showed similarity to that of *M*. *sediminis* YLB-01^T^, isolated from deep-sea sediment sample in the south-west Indian Ocean (Yu et al., 2013); *M. kunmingense* JXJ CY 27-2^T^, isolated as attached culture of *Microcystis aeruginosa* FACHB-905 (Maf) from Lake Kunming, southwest China (Xiao et al., 2022); and *M. invictum* DC-200^T^, isolated from homemade compost (Vaz-Moreira et al., 2009).

**Table 3:** Cellular fatty acid profile of strain ET2^T^ and closely related reference strains of the genus *Microbacterium*.

Fatty acid	1	2	3	4
C_16:0_	3.1	2.5	3.5	_
Iso-C_14:0_	1.0	0.2	0.3	_
Iso-C_15:0_	4.0	6.8	5.5	7.0
Iso-C_16:0_	**32.0**	9.5	9.7	12.1
Iso-C_17:0_	1.80	9.6	3.0	–
Anteiso-C_15:0_	**35.3**	27.1	52.0	48.6
Anteiso-C_17:0_	**22.8**	43.4	25.1	24.4

1, ET2^T^ (results from this study); 2, *Microbacterium kunmingense* JXJ CY 27-2^T^ (Xiao et al., 2022); 3, *M. invictum* DSM 19600^T^ (Vaz-Moreira et al., 2009); 3, *M. caowuchunii* ST-M6^T^ (Tian et al., 2021); * The values are percentages (w/w) of total fatty acids; –, not detected.Bold values are indicated to emphasis the difference between the strains.

The DNA G+C content of the new strain ET2^T^ is 69.4 mol% that is within the range for the family Microbacteriaceae (60–76 mol%) and for the genus *Microbacterium* (64–72 mol%; Evtushenko, 2012). Strain ET2^T^ could be distinguished from its closest relatives *M*. *kunmingense* JXJ CY 27-2^T^ and *M*. *invictum* sp. nov. DC-200T by not only phenotypic cultural characteristics but also chemotaxonomically (**Table 4**). The presence of ornithine as the cell-wall diamino acid instead of lysine, remarkably higher relative percentage of iso-C_16:0_ together with the lower content of anteiso C17:0, makes the strain stand out from the rest of *Microbacterium* species. *Мicrobacterium сaowuchunii* ST-M6 also contains ornithine but rhamnose, ribose, mannose, and galactose as cell-wall sugars, whereas *M. kunmingense* JXJ CY 27-2^T^ contains mannose, ribose, galactose, rhamnose, and arabinose, and ET2^T^ has rhamnose, fucose, glucose, and galactose.

**Table 4:** Root formation of kidney bean (*Phaseolus vulgaris*) cuttings treated with external auxins and microbial culture broth.

Sample	Rhizogenesis	
	Stem height (cm)	Number of roots per one cutting
Water control	0.6 ± 0.22	11.5 ± 0.71
IAA	3.3^a^ ± 0.40	30.5 ^a^ ± 3.32
IPA	2.4 ^a^ ± 0.30	22.0 ^a^ ± 3.00
ET2	6.4 ^a^ ± 0.52	68.3 ^a^ ± 8.40

The following concentration of the standard indoles was used: a), IAA (30 µg/mL) and IAM (30 µg/mL). Values are the mean of five replicates ± SD. ^a^ The treatments are significantly different from the control by Student’s t-test (P ≤ 0.01).

Comparing the phenotypic, chemotaxonomic, and phylogenetic characteristics of the closest *Microbacterium* species, strain ET2^T^ represents a novel species of the genus *Microbacterium* for which the name *Microbacterium albopurpureum* sp. nov. [type strain ET2^T^ (= VKPM Ac-2212 = VKM Ас-2998)] is proposed. *Microbacterium albopurpureum* (al.bo.pur.pu.′re.um. L. neut. adj. *albopurpureum* white and purple-colored, referring to the variations in the colour of colonies from white to purple).

Members of the phylum Actinomycetota (or Actinobacteria) are ubiquitous and are often reported as those inhabiting environments with extreme temperatures, pH, salinities, and water limitation (Verma et al., 2017; Yaradoddi and Kontro, 2021). The representatives of the genus *Microbacterium* are also shown to possess strong natural resistance to various stress factors. *Microbacterium* sp. 3J1 produces amounts of trehalose, melibiose, fructose, and glucose that enhances drought tolerance of the bacteria and plants inoculated with this strain during the drought periods (Vílchez et al., 2018).

*Microbacterium testaceum* B-HS2 is one of the strains with significant production of antioxidant enzymes (ascorbate peroxidase, superoxide dismutase, peroxidase, glutathione S-transferease, and catalase), which confer oxidative stress tolerance (Elahi et al., 2019). This capacity together with biosynthesis of carotenoids that are highly efficient quenchers of singlet oxygen and scavengers of other reactive oxygen species assume microbacteria resist solar UV radiation and oxidative damage from sunlight (Reis-Mansur et al., 2019). In genome of ET2^T^, we could detect the genes of isoprenoid (*idi*) and carotenoid (*ctrI*) biosynthesis, encoding isopentenyl diphosphate isomerase and multifunctional enzyme carotene (phytoene) desaturase, respectively (CP128170). However, the strain does not possess yellow or orange pigmentation, typical for many species of the genus *Microbacterium* that might be due to some default biosynthesis of the final carotenes. Nevertheless, the strain acquired the capacity to produce a purple-colored pigment that evidently substitutes the lack of carotenes under the stress conditions that facilitate the existence of the strain in the rhizoplane of the aerial orchid roots. Further studies of its nature and functions are needed to discover the role of the pigment.

Recently, we showed that *Chiloschista parishii* formed tight associations with cyanobacteria, mostly diazotrophic (Tsavkelova et al., 2022) that could supply nitrogen to the host plant under mineral-deficient conditions that might occur in tropical canopies at the altitude of 700 m up to 1,800 m above the sea level, where the plants usually grow (Teoh, 2021). The isolated ET2^T^ strain grew diazotrophically in the nitrogen-limited medium with increasing cell density up to 1.3 × 10^7^ CFU/mL after 4 days of cultivation in nitrogen-free medium with sucrose as the carbon, confirming its ability to fix nitrogen. *Microbacterium kunmingense* JXJ CY 27-2^T^ associated with cyanobacteria of *Microcystis aeruginosa* FACHB-905 and closely related to the described strain was also reported as a nitrogen-fixing bacterium. This ability provided an additional advantage to the establishment of successful partnership of *Microbacterium* with its plant or cyanobacterial macrohosts.

### Auxin production and identification of microbial indoles

**Figure 5:**
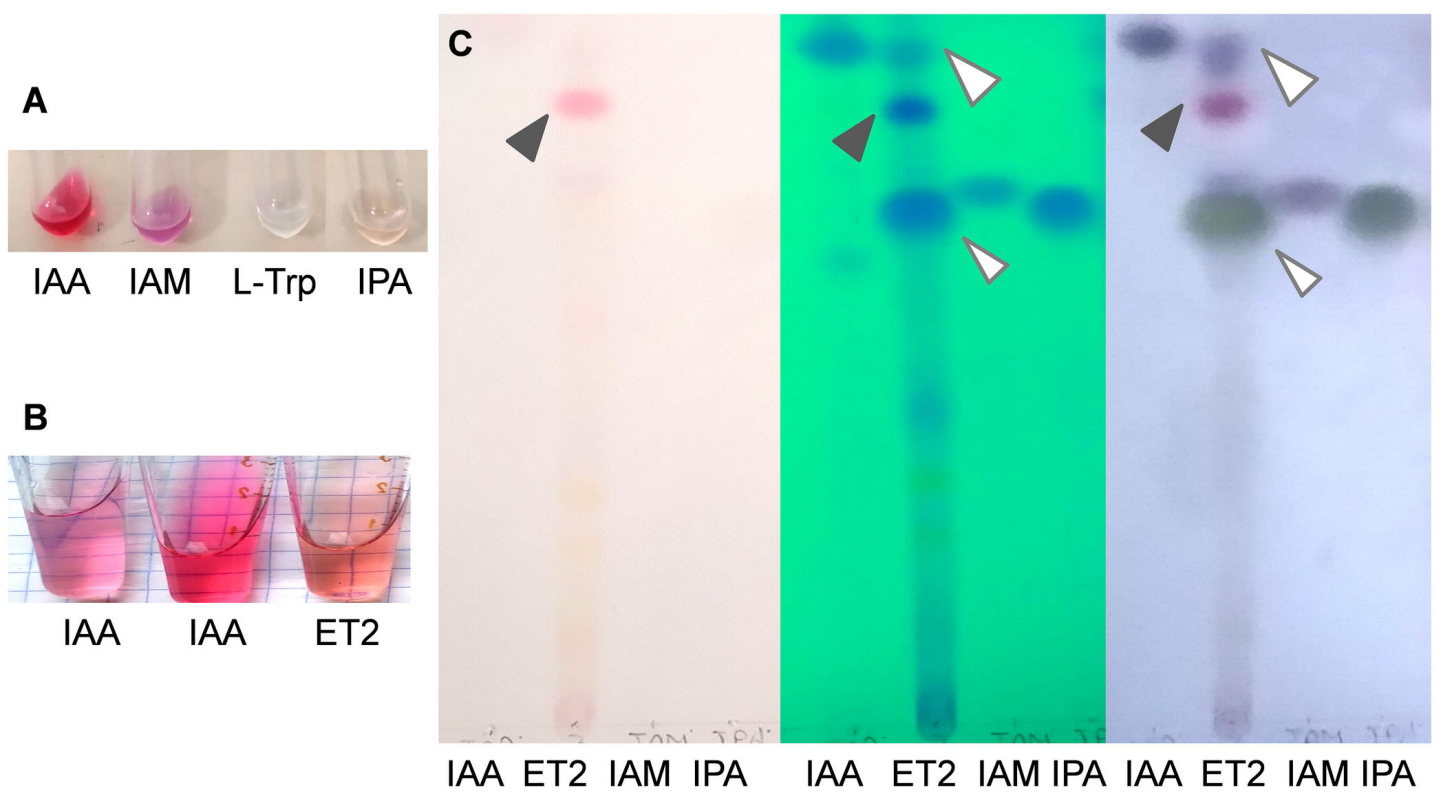
Identification of the auxins produced by *Microbacterium* ET2^T^. The colorimetric analysis of the standard auxins and L-tryptophan **(A)** and IAA and microbial auxins **(B)** with Salkowski regent. The nitrogen limited medium, supplemented with L-Trp (400 μg/mL) was used for cultivation of *Microbacterium* strain ET2. Total indoles were extracted with ethyl acetate and analyzed by TLC in chlorophorm:ethyl acetate:formiat (50:40:10) running solvent. The plates **(C)** from left to right are as follows: the plate removed from the tank (the pink spot refers to the pigment); the plate placed under the UV-light (indoles are visualized under UV_254_); and the plate after spraying of the reagent and heating (indoles acquire blue-violet color of different intensity. The standards are designated as IAA, indole-3-acetic acid; IAM, indole-3-acetamide; IPA, indole-3-pyruvic acid; and L-Trp, L-tryptophan. The following concentrations were used: **(A)** IAA (2 mM), IAM (1 mM), L-Trp (1 mM), and IPA (1 mM); **(B)** IAA (30 μg/mL) and IAA (80 μg/mL); and **(C)** IAA (2 mM), IAM (1 mM), and IPyA (1 mM).

The revealed activities of ET2^T^ could implicitly refer to its production of diverse secondary metabolites, including phytohormones that turn to be a sort of communication opportunity and strategy between the host plant and its associated PGPR. It is particularly applicable to auxins, which major and direct impact affects the root formation and growth. Under exogenous L-tryptophan supplementation, the strain ET2^T^ cultivated in nitrogen-free medium, showed a prominent ability to produce auxins. Estimated by staining with Salkowski reagent, it produced 24.3 ± 1.77 µg/mL. Considering the biomass accumulation (OD_540_) during 4 days of incubation up to 0.8 ± 0.17, relative levels of auxin yield reached 30 µg per OD unit. Salkowski reagent is not specific for IAA, and it also allows to detect indole-pyruvic acid (IPA) and indole-acetamide (IAM) (Glickmann and Dessaux, 1995; Patten and Glick, 2002), whereas tryptophan gives no coloration (**Figure 5**). Although the above-mentioned indoles are detectable with the reagent, IPA and IAM compounds give different shades of peach and violet, respectively, whereas IAA is colored pink. Thus, the final color may vary depending on the presence and amount of different indoles. Auxins produced by strain ET2^T^ resulted in more peach pink color than intense pink of the pure IAA solution, implying the mixed nature of the produced auxins (**Figure 5B**).

In order to determine the ability of the strain to synthesize IAA *de novo*, we used TLC to detect the key intermediates, such as indole-3-acetamide (IAM pathway) and indole-3-pyruvic acid (IPyA pathway), which constitute the major sources of common microbial auxin production. The results of TLC analysis confirmed the data of relative auxin estimation by colorimetric assay; apart from IAA, a big blot corresponding to IPyA was revealed (**Figure 5C**). The amounts of IPyA were surprisingly much higher than those of IAA being the final product. That might be due to the non-active conversion of IPyA to indole-3-acetaldehyde by indole-3-pyruvate decarboxylase. Additionally, a conjugate formation that could also influence the total amount of the auxins should be also taken into account, when free IAA can be rendered inactive by conjugation to sugars or amino acids (Duca and Glick, 2020). Although such conjugates were observed not within PGPR group but mainly in pathogenic bacteria, including *Pseudomonas savastanoi* strains, converting IAA into IAA–Lysine conjugates to deliver continuous levels of IAA to host tissues (Duca and Glick, 2020; Tegli et al., 2020).

No determinants for the IAM pathway have been detected, either by UV-light detection or by visualization with van Ehmann’s reagent. Other potential spots were hardly detected, except for the initially pink one. This blot can be potentially referred to the purple pigment, which could also be extracted by ethyl acetate. Further investigations are needed though for more precise and quantitative analysis of the intermediates, produced by ET2, for better understanding of the mechanisms underlying the production and regulation of auxin biosynthesis, as well as interactions with the plants.

Auxin biosynthesis has been previously reported for certain strains within the genus *Microbacterium*. We were the first who paid attention to the capacity of *Microbacterium* strains for auxin production (Tsavkelova et al., 2007b), reporting on the orchid-associated *Microbacterium* sp. D-23 strain (GenBank accession number AM498042.1). It produced IAA via indole-3-pyruvic pathway, corresponding to the intermediates of IPA and IAAld, detected by HPLC. Remarkably, the strain *Microbacterium* sp. D-23 also demonstrated disproportion in produced auxins with much higher amounts of the precursors than the final product. Currently, D-23 strain shows the closest similarity to *Microbacterium testaceum* strain SK3-22.2.1B (MN421065.1) that correlate with another recent report of Yadav et al. (2022). The recent research postulates that *Microbacterium testaceum* Y411 (MZ367021.1) isolated from another epiphytic orchid, *Rhynchostylis retusa* (L.) Blume., is a strong producer of IAA. It managed to form IAA (158.62 µg/mL) but required significant tryptophan supplementation of 1000 µg/mL for the IAA biosynthesis. More than half of the isolates (59% out of 70 *Microbacterium* belonging to 20 different species) were able to produce IAA via tryptamine pathway based on the RAST database. They include *M. azadirachtae* ARN176, *M. hydrocarbonoxydans* SA35, and *M. oxydans* BEL163 initially isolated from heavy metal contaminated soil, plant rhizosphere, and *Salix viminalis* roots, respectively, as well as from *M. ginsengisoli* DSM 18659 and *M. trichothecenolyticum* DSM 8608 isolated from the non-contaminated soils (Corretto et al., 2020). Thus, plant-associated *Microbacterium* species appear to be among the most beneficial PGPB.

### Biological activity of the auxins

**Figure 6:**
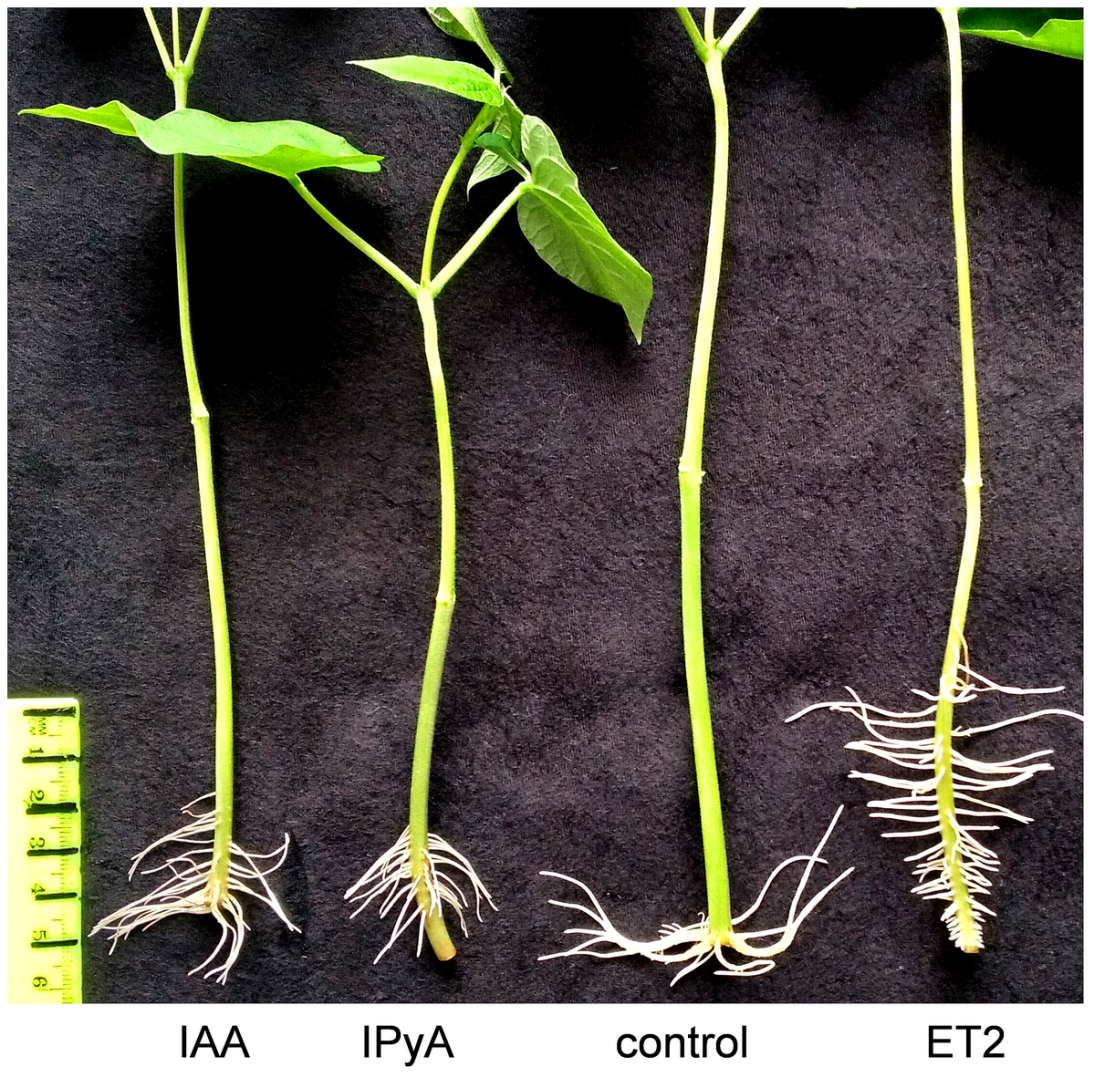
Rooting of the kidney bean (*Phaseolus vulgaris* L.) cuttings. The cuttings were submerged in water (control), IAA of 30 µg/mL, and culture broth (supernatant) of *Microbacterium* ET2^T^. The data presented 10 days after treatment.

Auxin is the main plant growth stimulator, which directly influences and accelerates root formation and growth. The bioassay performed with bean cuttings deprived of their roots showed a considerable promotion of rooting by microbial auxins produced by strain ET2^T^ (**Figure 6**, **Table 5**). The height of the root formation and the root number were 10.6 and 5.9 times higher than in the control group, respectively. Moreover, the rhizogenesis promotion was much more evident for the samples treated with microbial culture broth than for the IAA-treated cuttings, confirming the synergic influence of other indoles. We showed that exogenous IPA could also stimulate the formation and root development, just 1.4 times less then IAA of the same concentration (30 µg/mL). However, to investigate the effects and influence of diverse bacteria-derived auxins on the plant rhizogenesis, further investigations using the knockout mutants are needed.

**Table 5:** Putative genes of strain ET2^T^ involved in biosynthesis of indole-3-acetic acid.

Enzyme	Presence in ET2^T^ genome	Baits	Identity (%)
IAN pathway (indole-3-acetonitrile, indole-3-acetamide)			
Aldoxime dehydratase	No hits	XP_003716984.1, *Pyricularia oryzae* 70-15 Q76K71, *Rhodococcus erythropolis* A0A3D9E9G7, *Microbacterium* sp. AG157 Q4W3E6, *Rhodococcus erythropolis*	–
Nitrilase	WJL94913.1 and WJL94547.1	According to the annotation in ET2^T^ genome	–
Nitrile hydratase	No hits	Q4W3F2, *Microbacterium* sp. AJ115	–
Indole-3-acetamide hydrolase	WJL95197.1	P19922, *Bradyrhizobium japonicum* AB025110.1, *Agrobacterium tumefaciens*	33%
Indole-3-acetamide			
tryptophan 2-monooxygenase	No hits	P25017, *Agrobacterium vitis* P0A3V3, *Rhizobium radiobacter* Q47861, *Pantoea agglomerans*	–
Indole-3-acetamide hydrolase	WJL95197.1	P19922, *Bradyrhizobium japonicum* AB025110.1, *Agrobacterium tumefaciens*	33%
Indole-3-pyruvic acid to Indole-3-acetaldehyde			
Amino transferase	WJL95463.1 WJL96360.1	AHZ17137.1 *Bacillus velezensis* SQR9 XP_003715165.1 *Pyricularia oryzae* 70-15	32%, 30%
Indole-3-pyruvate decarboxylase	WJL96591.1	P9WG37, *Mycobacterium tuberculosis*	56%
Indole-3-acetaldehyde dehydrogenase	WJL97005.1	V529_39560, *Bacillus velezensis* SQR9	33%
Tryptamine			
Tryptophan decarboxylase	No hits	A7B1V0, *Ruminococcus gnavus* J7SZ64, *Clostridium sporogenes*	–
Amine oxidase	No hits	P80695, *Klebsiella oxytoca* P46883, *Escherichia coli*	–
YUCCA Pathway			
Flavin monooxygenase	No hits	NP_193062.1, *Arabidopsis thaliana* NP_171955.1, *Arabidopsis thaliana*	–

The genes were mined by using protein functions in annotation of the ET2 whole genome, as well as by blasting sequences of bacterial IAA-related proteins as queries in UNIPROT and NCBI databases (see Materials and Methods).

Microbial auxins directly influence plant rooting for the mutual benefit of consortial interactions. IAA acts as a cross-talk molecule by enhancing plant rhizogenesis and reciprocally establishing new sites for PGPB populations upon their consuming of the root exudates from the newly formed roots (el Zahar Haichar et al., 2014). We also repeatedly demonstrated that PGP bacteria associated with both greenhouse (Tsavkelova et al., 2016) and wild grown (Tsavkelova et al., 2007a) orchids may significantly promote plant rhizogenesis. The bioassay with *Bacillus*, *Erwinia*, and *Burkholderia* isolated from *Paphiopedilum appletonianum* (Gower) Rolfe. and *Flavobacterium* and *Pseudomonas* isolated from the epiphytic *Pholidota articulata* Lindl. orchids showed their rhizogenesis activity and established them as comparable to exogenous IAA of a concentration of 50 µg/mL (Tsavkelova et al., 2007a). In this study, we demonstrate the important effect of microbial indole-3-pyruvic acid that may significantly add to the IAA content in the cultural broth and thus to the ultimate growth-promoting effect of the strain ET2^T^.

### Identification of putative genes involved in IAA biosynthesis in ET2 genome

The main microbial IAA biosynthetic pathways are tryptophan-dependent (Tsavkelova et al., 2006; Spaepen and Vanderleyden, 2011); the most functional routs, according to the key intermediate compound, are indole-3-pyruvic acid (IPyA), indole-3-acetamide (IAM), indole-3-acetonitrile (IAN), and tryptamine (TAM) pathways. Two others, such as tryptophan side-chain oxidase (TSO) and flavin monooxygenase (YUCCA) pathways, which were found in plants, also reported to occur in root-associated bacteria, much more rare though.

Based on the identification of the final products and the major intermediates, we also searched for the putative genes of IAA biosynthesis via IPA, IAM, IAN, TAM, and YUCCA pathways in the genome of ET2^T^ (CP128170). The putative genes were screened considering their deduced protein domains, which were reported as functional in IAA metabolism (**Table 1**). According to the obtained results, the major contributor to IAA biosynthesis is IPA pathway. We could identify a complete (IPA)–based pathway, consisting of the putative genes for aminotransferase(s), converting tryptophan to IPA, indole-3-pyruvate decarboxylase (IPDC), converting IPA to IAAld, and indole-3-acetaldehyde dehydrogenase, involved in the final step of turning IAAld to IAA. This route seems to be the only functional because others like IAN or IAM pathways are most likely incomplete, or as it was shown for YUCCA or tryptamine pathways, absent at all.

These results perfectly correlate to our data on auxin compound identification, which showed the accumulation of IPA with no significant amounts of other intermediates. The homologous of the characterized IPDC was revealed in *Mycobacterium tuberculosis* (P9WG37) only with its 56% identity, but we could not identified IPDC enzymes established in other known PGPR bacteria. This might be a reason for some deviations in IPDC functioning resulted in IPA accumulation and its less efficient conversion to the final product. Further investigations are needed though, to characterize thoroughly the activity of IPDC enzyme in ET2 strain.

### Plant growth-promoting capacity

**Figure 7:**
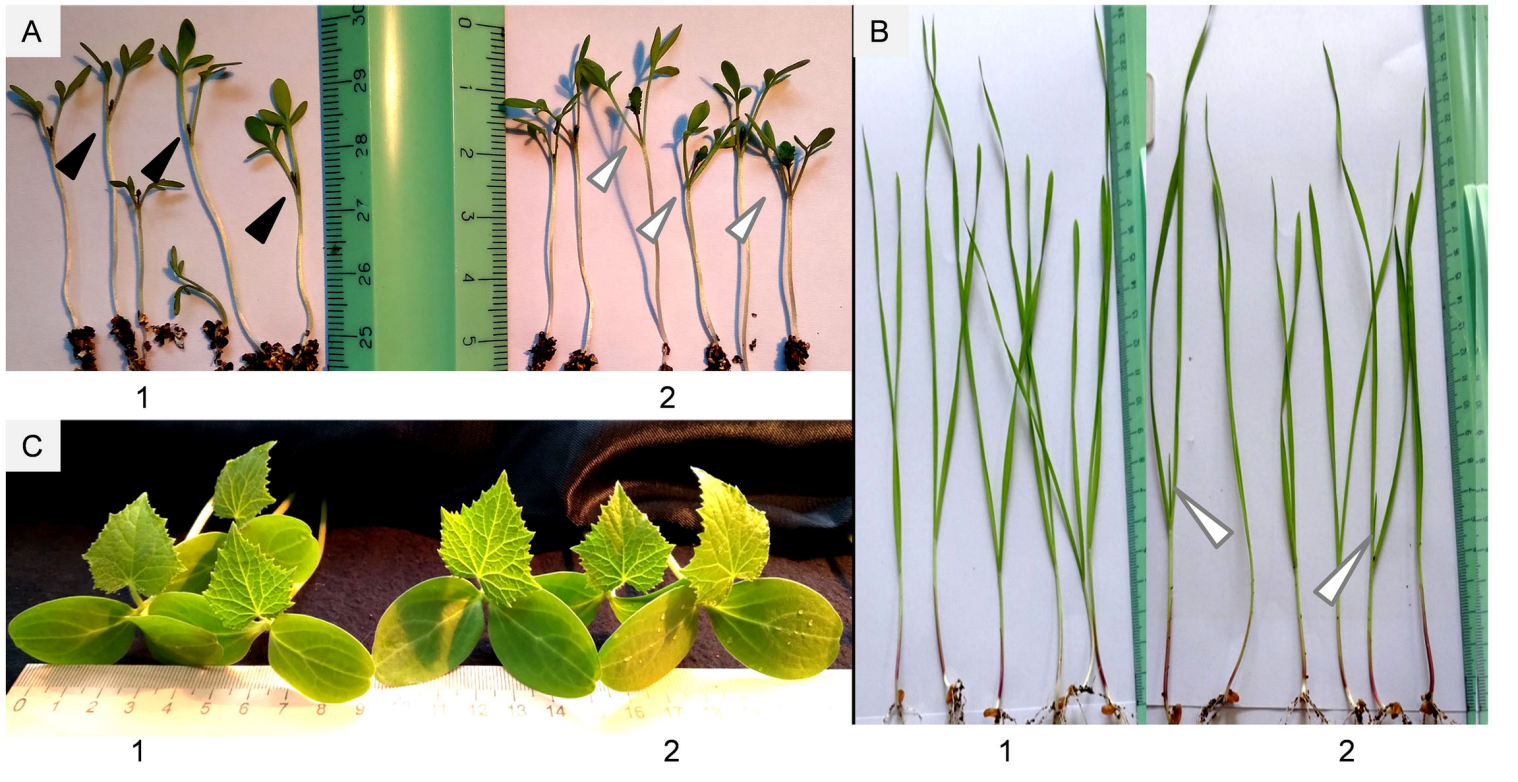
Plants inoculated with *Microbacterium* ET2^T^: **(A)** garden cress (*Lepidium sativum* L.); **(B)** wheat (*Triticum aestivum* L.); **(C)** cucumber (*Cucumis sativus* L.). The plants are represented with control (1) and bacterized with ET2 (2) variants at 14 **(A)**, 16 **(B)**, and 18 **(C)** days after inoculation, respectively. A third leaf rudiment is indicated with black arrows, and a well-developed third leaf is indicated with white arrows.

**Figure 8:**
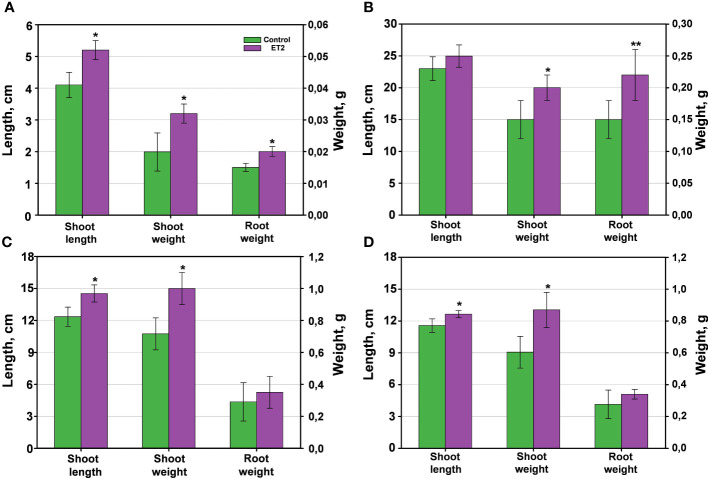
The plant growth-promoting capacity of *Microbacterium* ET2^T^. The plants are **(A)** garden cress (*Lepidium sativum* L.), **(B)** wheat (*Triticum aestivum* L.) and **(C, D)** cucumber (*Cucumis sativus* L.) under the cold-stress treatment at 4°C for the first 24 h of the development **(C)** and normal temperature **(D)** conditions. The parameters are the length of the aerial vegetative parts (shoot length, cm), the biomass of stem and leaves (shoot weight, g), and the biomass of the roots (root weight). The values are the average per variant ± SD. Asterisks (* and **) indicate the significant difference between the control and inoculated plants at the level of p < 0.05 and p < 0.1, respectively.

Considering high efficiency of *Microbacterium* ET2^T^ auxins on the rhizogenesis, bacterization of several agriculture plants (wheat, cucumber, and garden cress) were studied. Treatment of the seeds with ET2^T^ resulted in increased lengths and biomass of the vegetative parts and roots of the crops (**Figures 7**, **8**). ET2-treated garden cress was 27% (*P* < 0.05) higher than the control group. The plants had accelerated growth due to formation and development of the third leaf clearly visible in the treated seeds but absent or scarce in the control group (**Figure 7**). Altogether, the total biomass of the bacterized plants of *Lepidium sativum* was 1.5 and 1.3 times higher for the aerial and underground parts, respectively, than those of the untreated plants. Growth of wheat plants was also promoted by ET2^T^ (**Figure 8B**); vegetative biomass production was significantly (*P* < 0.05) higher than that of the control plants. The enhancement in other growth parameters was also recorded (0.05 < *P* < 0.1), although not significantly different (**Figure 8B**).

Stimulation of cucumber growth under ET2^T^ treatment was also observed. The green aerial parts (shoot height and biomass) of the treated plants under the cold-stress conditions (**Figure 8C**) were significantly (*P* < 0.05) higher compared to the untreated plants. Moreover, the development of the aboveground parts was even more pronounced when the plants were subjected to cold stress than those cultivated under normal conditions (**Figure 8D**). The bacterization clearly induced the height and improved the weight gain of cucumber plants. The stressed plants responded more strongly and positively with 17.4% longer stems and 35.7% increased biomass, including larger leaf blades (**Figures 7C**, **8C**), compared to the untreated plants.

The obtained results highlight the evident beneficial properties of ET2. Its auxin-producing capacity together with additional ability to fix nitrogen directly promotes the growth and adaptation of the treated plants, particularly under cold stress environmental conditions.

PGP activity of root-associated bacteria is well known and widely applied for the bacterization or bio-fertilization of diverse crops. *Microbacterium* strains also express their favorable impact on agriculture plant growth and development in quite a various directions, indicating their prospective. *Microbacterium imperiale* (MAIIF2a) increased cassava (*Manihot esculenta*) biomass and mitigated root rot in cassava as a biocontrol agent against fungal pathogen, *Fusarium solani* (Freitas et al., 2019). *Microbacterium aurantiacum* was shown to promote *Arabidopsis* growth through auxin, salicylic acid, and gibberellin production as well as via predicted by transcriptome analysis the biosynthesis of volatile organic compounds, which promoted tobacco growth (Gao et al., 2022). Endophytic *Microbacterium* sp. (BXGe71) isolated from *Arabis alpine* significantly enhanced host plants’ shoot lengths, root lengths, and dry weights under multi-heavy metal stress conditions (Sun et al., 2019) as well as another Pb-resistant strain of *Microbacterium* sp. G16, which was reported to increase rape (*Brassica napus*) biomass in Pb-added soils, due to its capacity of auxin biosynthesis, siderophore production, and formation of ACC deaminase, reducing ethylene level (Sheng et al., 2008). The most closest to ET2, JXJ CY 27-2^T^ isolate of *M. kunmingense* promoted the development of its co-culture partner, cyanobacteria *Microcystis aeruginosa* FACHB-905, when inoculated in modest amounts of 1 × 10^5^ CFU/mL, supplying it with available phosphorus, nitrogen, and other nutrients such as vitamins and auxin, as predicted from Gene Ontology (GO) annotation database (Xiao et al., 2022).

Thus, the accumulating data on the evident and efficient promotion of different plant growth under normal and stressful environmental conditions make the root-associated colonizers of the genus *Microbacterium* one of the most helpful and indispensible partners to better host plant resistance, adaptation, and competitive advantage.

### Species description of ET2^T^

*Microbacterium albopurpureum* (al.bo.pur.pu.′re.um. L. neut. adj. *albopurpureum* white and purple-colored, referring to the variations in the colour of colonies from white to purple).

Cells are Gram-positive non-motile rod-shaped (0.8-µm to 1.4-µm long and 0.3-µm to 0.5-µm wide) with aerobic lifestyle. Colonies are slow-growing, whitish or slightly pink pigmented, convex, up to 1.5–2.5 mm in diameter, and circular after 4–7 days of incubation in R2A medium at 30°C. Growth occurs in R2A and TSA media. Colonies turn bright purple with time or under competitive biotic conditions. Growth occurs at temperature between 25°C and 55°C (optimal at 30°C−37°C), pH 6.0–9.0 (optimum, pH 7.0) tolerating NaCl up to 10% (optimal at 0.5% NaCl). Catalase test is positive. Weak positive reaction for glycerol assimilation and starch hydrolysis. Negative for nitrate reduction, assimilation of trisodium citrate and pyruvate, for the hydrolysis of gelatin and casein. Acid is produced from sucrose, galactose, D-mannose, D-maltose, and D-mannitol. Cell-wall peptidoglycan contains L-ornithine, alanine, valine, glutamic acid, and glycine. Cell-wall sugars contain glucose and galactose as the main sugars and fucose with rahmnose as minor sugars. Predominant cellular fatty acids are anteiso-C_15:0_ (35.35%), iso-C_16:0_ (31.96%), and anteiso-C_17:0_ (22.78%); the minor components include iso-C_15:0_ (4.0%), C_16:0_ (3.08%), iso-C_17:0_ (1.80%), and iso-C_14:0_ (1.02%). The polar lipids consist of phosphatidylglycerol and diphosphatidylglycerol, unidentified glycolipids, and polar lipid. The DNA G+C content based on complete genome sequence is 69.4%.

The type strain, ET2 (=VKPM Ac-2212=VKM Ас-2998), was isolated from the aerial roots of leafless epiphytic orchid, *Chiloschista parishii* Seidenf. The whole-genome sequence of ET2 is deposited in GenBank (CP128170).

## Data availability statement

The datasets presented in this study can be found in online repositories. The names of the repository/repositories and accession number(s) can be found in the article/supplementary material.

## Author contributions

ET: Conceptualization, Data curation, Formal analysis, Investigation, Methodology, Project administration, Supervision, Validation, Visualization, Writing – original draft, Writing – review & editing. EV: Writing – original draft, Writing – review & editing, Investigation, Software. NP: Writing – original draft, Writing – review & editing, Investigation, Methodology. KL: Writing – original draft, Writing – review & editing, Investigation, Software. AA: Writing – original draft, Writing – review & editing, Investigation, Methodology.
